# Supplementary material for: Evaluation of anticancer potential of tetracene-5,12-dione (A01) and pyrimidine-2,4-dione (A02) via caspase 3 and lactate dehydrogenase cytotoxicity investigations
Source: PLoS One. 2023 Dec 21;18(12):e0292455. doi: 10.1371/journal.pone.0292455 (PMC10734984; doi:10.1371/journal.pone.0292455)
Supplement: S1 File — (DOCX) [file pone.0292455.s001.docx]

**Evaluation of Anticancer Potential of Tetracene-5,12-Dione (A01) and Pyrimidine-2,4-Dione (A02) via Caspase 3 and Lactate Dehydrogenase cytotoxicity investigations**

Mubashir Aziz^1^, Muhammad Sarfraz^2^, Muhammad Khurrum Ibrahim^3^, Syeda Abida Ejaz^1*^, Tasneem Zehra^4^, Hanan A. Ogaly^5^, Mosab Arafat^2^, Fatimah AM Al-Zahrani^5^, Chen Li^6*^

*^1^ Department of Pharmaceutical Chemistry, Faculty of Pharmacy, The Islamia University of Bahawalpur, Bahawalpur 63100, Pakistan*

*^2^College of Pharmacy, Al Ain University, Al Ain, 64141, United Arab Emirates*

*^3^Baqai Medical University Karachi 75340, Pakistan*

*^4^Department of Basic Science & humanities, Dawood University of Engineering & Technology, Karachi, 74800, Pakistan*

*^5^Chemistry Department, College of Science, King Khalid University, Abha 61421, Saudi Arabia. ^6^Department of Biology, Chemistry, Pharmacy, Free University of Berlin, Berlin 14195, Germany*

**Corresponding Authors**

**Syeda Abida Ejaz:** [**abida.ejaz@iub.edu.pk**](mailto:abida.ejaz@iub.edu.pk)

**Chen Li:** [**chen.li@fu-berlin.de**](mailto:chen.li@fu-berlin.de)

**2. Experimental**

***2.1. In-vitro Studies***

**2.1.1. Cell viability Assay**

Initially the compounds **A01 and A02** were tested for its anticancer potential against two different cell lines;  human breast cancer cell lines (MDA-MB231; ATTC: HTB-26™ and MCF-7 cell line; ATTC: HTB-22™) and one human cervical cancer cell line (HeLa; ATTC: CRM-CCL-2™). The effect was also observed against normal cell lines, i.e., African green monkey kidney (Vero) cells (ATTC: CCL-81™). The experiment was performed by doing the slight modifications in the already reported method of Mosmann (in 1983) and Nikš and Otto (in 1990), respectively [23, 24]. The experiment was performed in 96–well flat–bottom plates in which 90 μL of medium containing 10 × 10^4^ cells were seeded into each well. The 100 µL of test compound solution was added to the respective well and the plate was allowed for 24 hours of incubation at 37 ˚C and 5% CO2. The positive and negative control wells were seeded with10 μL of standard drugs (doxorubicin and cisplatin) and 100 μL of cells media (no compound), respectively. Each well was then pipetted with 10 μL of MTT reagent and incubated for 4 h at 37°C. Then 100 µL of 10% sodium dodecyl sulphate solution was added and kept at room temperature for 30 min. with occasional shaking. Finally, optical density was calculated. The ability of mitochondrial dehydro-genase to generate formazan complex indicated the presence of metabolically active cells (viable cells). All experiments were carried out in triplicate and results were reported as percent growth inhibition values as reported earlier [28].

**2.1.2. Lactate Dehydrogenase Cytotoxicity Assay**

Lactate dehydrogenase is an enzyme present in cytosol of almost all the cells and is released in extracellular medium upon membrane rupture. LDH assay was performed to assess cytotoxic potential of **A01** and **A02** against HeLa, MDA-MB-231 and MCF-7 cells. Amount of FBS was reduced to 3% in complete culture medium while 10 x 10^3^ cells were optimized per well for this assay. Cytotoxicity of **A01** and **A02** was tested against HeLa, MDA-MB-231 and MCF-7 cells at its respective IC_50_ and 2x IC_50_ values in these cells using method as mentioned in literature [29] and as per protocol mentioned in LDH Assay Kit (Cytotoxicity; ab65393). The absorbance was carried out at CLARIOstar Plus microplate reader (BMG Labtech, Germany).

**2.1.3. Apoptosis assessment by caspase 3 activity**

Caspase-3 activity was analyzed using a Caspase-3 Assay Kit (Fluorometric:ab39383). The cell (HeLa, MDA-MB-231 and MCF-7 cells) were plated at 1 × 10^6^ cells in 6 well plate in 2 mL of media overnight. The cells were treated with **A01** and **A02** using concentration equivalent to the IC_50_ and 2x IC_50_ values obtained during MTT assay, using method as mentioned in literature [30] and as per protocol mentioned in Caspase-3 Assay Kit (Fluorometric:ab39383). Adherent cells were collected, centrifuged, and lysed using 50 μL of lysis buffer on ice for 10 min, and incubated with DEVD-AFC substrate for caspase-3 [31] followed by the addition of reaction buffer at 37 °C for 3 h. After incubation of a substrate with a cell lysate sample, the amount of fluorescent cleavage product was measured using the CLARIOstar Plus microplate reader (BMG Labtech, Germany). All these experiments were performed in triplicate.

**Molecular Docking**

**Validation of docking protocol**

To validate the docking protocol, we performed redocking of the co-crystal ligand RXB with the caspase 3 enzyme. The native and redocked poses of the co-crystal ligand are represented by green and pink colors, respectively. The RMSD value of the redocked pose was found to be less than 2 angstroms (1.8 angstroms), indicating the successful validation of our docking protocol.


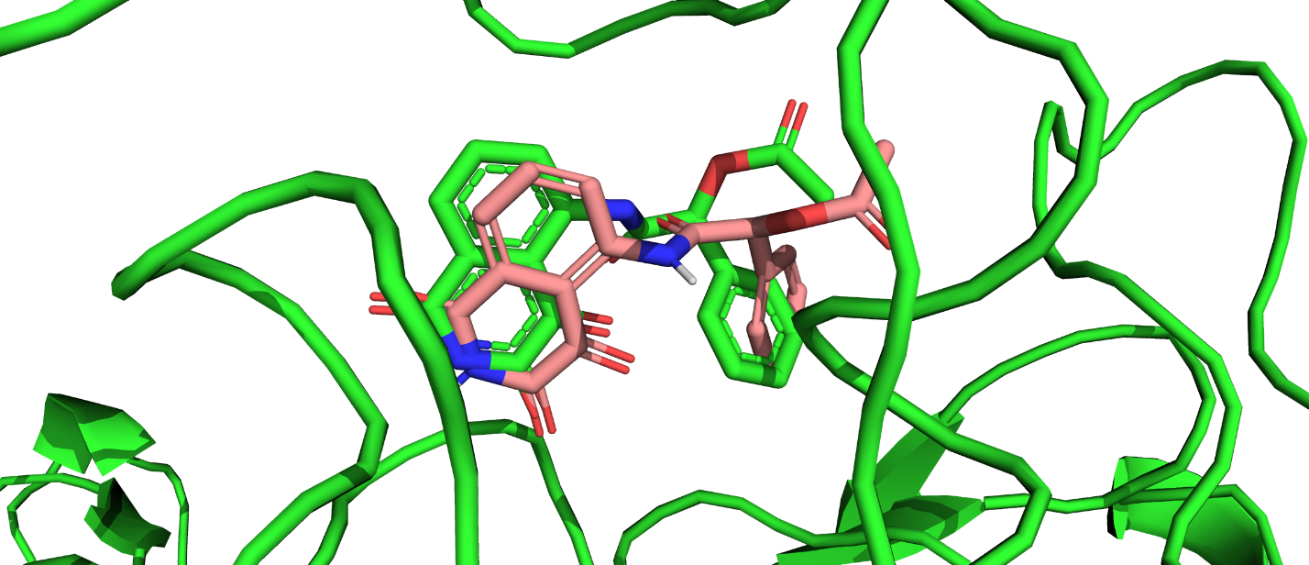


**Figure S1**. Redocking of co-crystal ligand RXB with caspase 3 enzyme. Green colored ligand indicate the native pose whereas pink colored indicate the redocked pose of co-crystal ligand. The RMSD of less than 2 angstroms (1.8 angstroms) indicate the validation of docking protocol.

***In vitro* Cytotoxic Activity**

**GI_50_ graph**

**
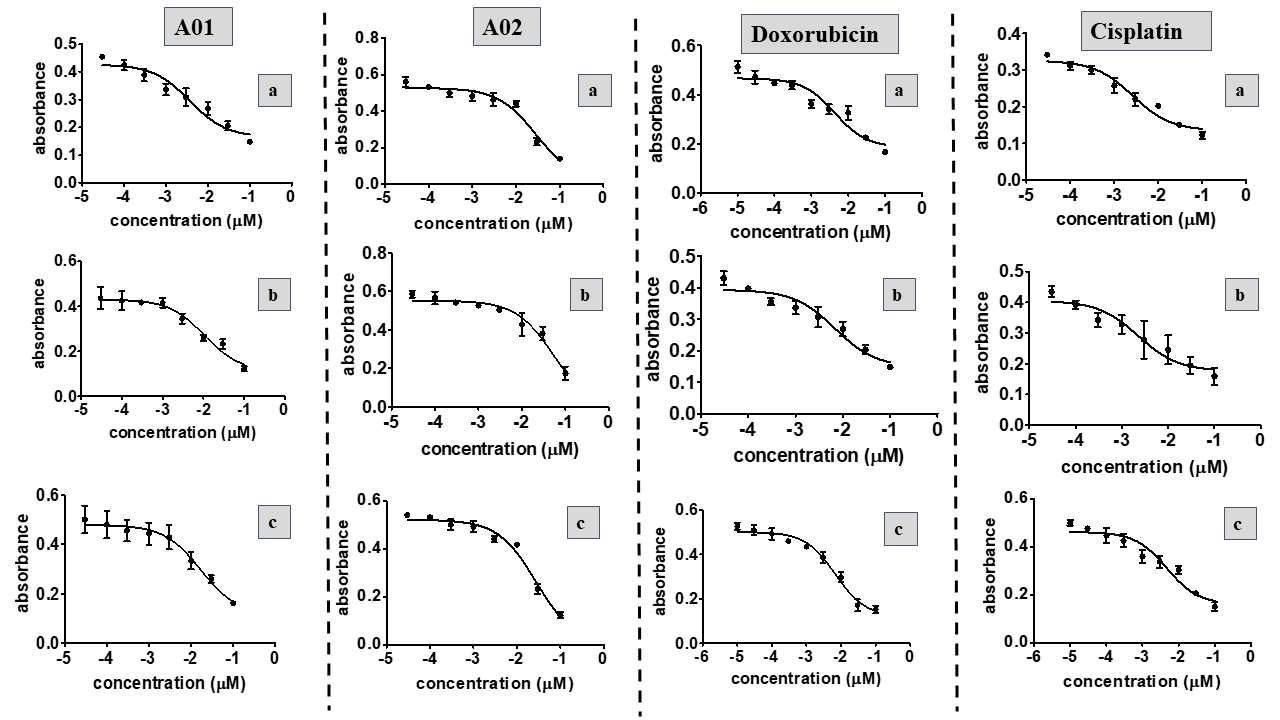
**

**Figure S2.** GI_50_ graph of **A01**, **A02**, Doxorubicin and Cisplatin against a) HeLa, b) MDAMB-231 and c) MCF-7 cells

**Molecular Dynamics Simulations**

A molecular dynamics simulation was conducted to examine the interaction between caspase 3 protein and compounds **A01** and **A02**, spanning a simulation time of 100 ns for each complex. Notably, both complexes exhibited consistent and stable trajectories, showing clear equilibration trends over the entire 100 ns duration. The calculated average root-mean-square deviation (RMSD) values for the caspase 3-**A01** and caspase 3-**A02** complexes were 3.5 angstroms and 3.2 angstroms, respectively. This suggests that the complexes maintained their structural stability throughout the simulation period. Furthermore, the ligands (**A01** and **A02**) displayed sustained attachment to the active site residues of caspase 3, as illustrated in Figure S3. This visualization underscores the robustness of the ligand-protein interactions and supports the notion that both compounds maintain their binding configurations within the active site region throughout the simulation. The findings from this molecular dynamics simulation provide valuable insights into the dynamic behavior of the caspase 3 complexes with **A01** and **A02**, reinforcing the stability and relevance of their interactions at the molecular level.


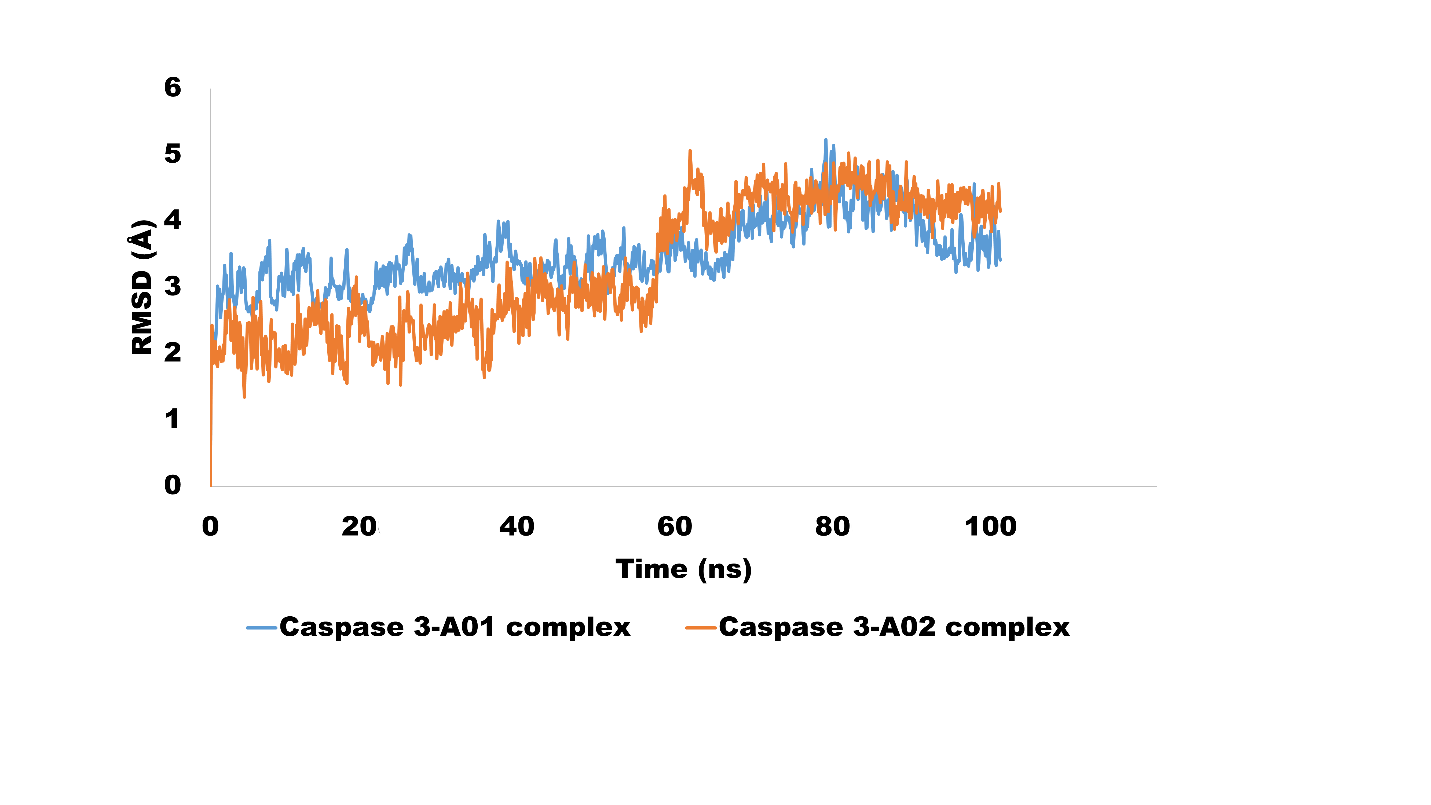


**Figure S3.** RMSD plot of **Caspase3-A-01 complex** and **Caspase3-A-02 complex**

**Molecular Interactions of standard drugs**

In order to provide direct comparison of molecular docking, standard compounds were docked with respective bio targets alongside A01 and A02. Both compounds scored better than standard drugs as interactions are provided in table S1.

**Table S1. Molecular interactions exhibited by standard molecules against Caspase 3, P53 and Kappa protein**

| **Complex** | **Binding energy (kcal/mol)** | **Hydrogen bonding** | **Hydrogen bond length (Å)** | **Hydrophobic interactions residues** |
| --- | --- | --- | --- | --- |
| P53-Doxirubicin | -10.53 | Asn17, Gln23, Arg10 | 2.69, 3.20, 3.18 | Glu89, Ile22, Lys20, Tyr92, Cys114, Arg203, Pro231 |
| NF-κB-Doxorubicin | -4.52 | Thr682, Arg685 | 2.94, 2.93 | Pro681, Thr740 |
| Caspase 3-Doxorubicin | -9.49 | His121, Gly122, Leu168 | 3.24, 2.2, 2.96 | Leu168, Phe256, Thr166, Thr255 |
| DNA-Doxorubicin | -9.93 | Dt20, Dt20, Dt19, Dt8, Dt7 | 2.77, 3.26, 3.10, 2.66, 3.22 | Da6, Dc21, Da5, Dg4, Dg22 |
| P53-cisplatin | -3.27 | Asn17, Ile21, Phe16 | 3.34, 2.98, 3.32 | Ly20, Ile22, Gln23 |
| NF-κB-cisplatin | -2.38 | - | - | Leu674, Jis673, Leu736, Asn698, Asn669 |
| Caspase 3-cisplatin | -2.57 | - | - | Phe256, Thr255, Tyr204, Leu168, Phe256 |
| DNA-cisplatin | -3.79 | Dt8, Dt19, Dt7 | 2.92, 3.12, 2.96 | Dt20, Da6, Dc21 |
