# Supplementary figures and images for: Evaluation of anticancer potential of tetracene-5,12-dione (A01) and pyrimidine-2,4-dione (A02) via caspase 3 and lactate dehydrogenase cytotoxicity investigations
Source: PLoS One. 2023 Dec 21;18(12):e0292455. doi: 10.1371/journal.pone.0292455 (PMC10734984; doi:10.1371/journal.pone.0292455)

**Graphical Abstract**


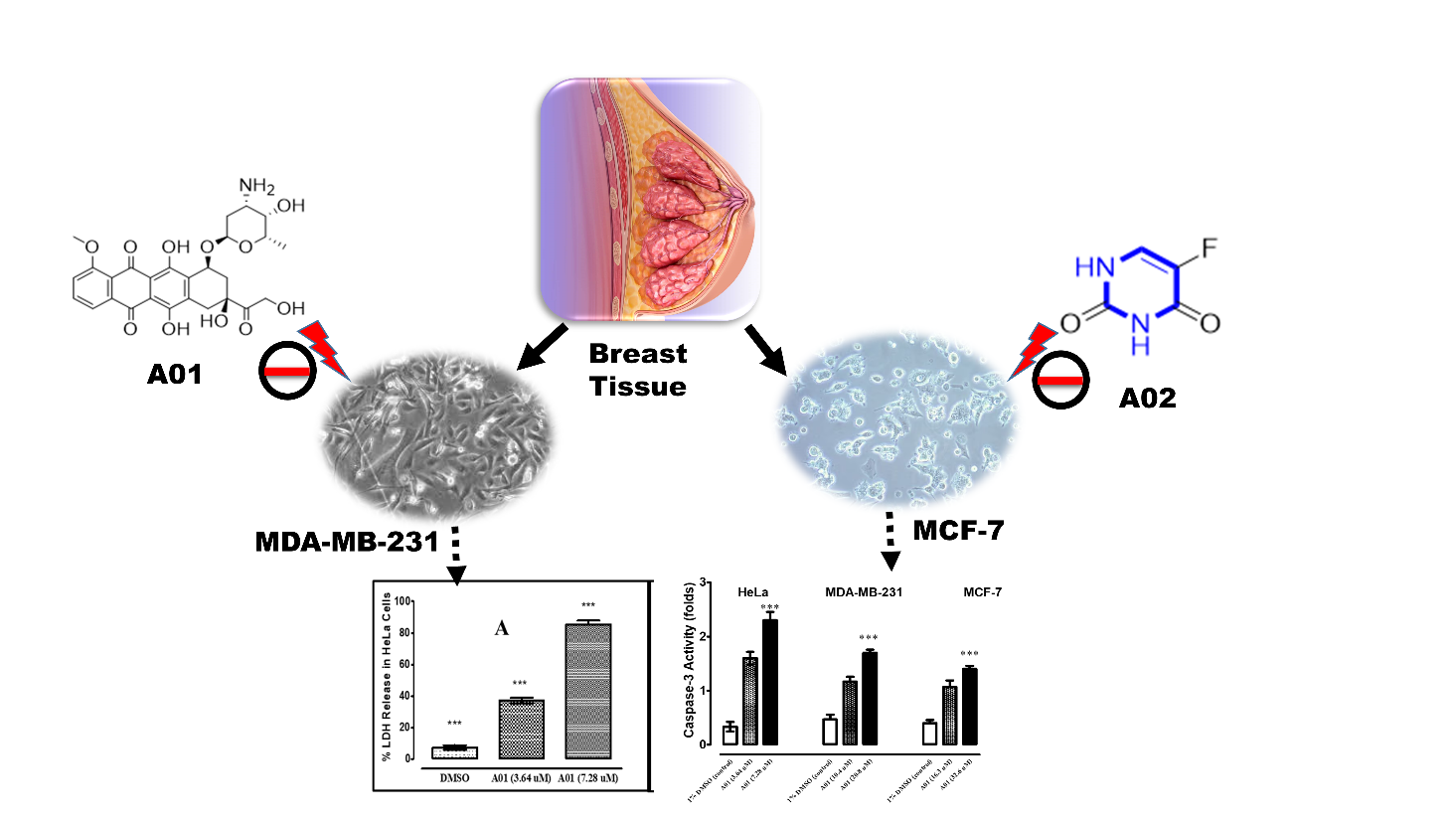

Supplement: S1 Graphical abstract — (DOCX) [file pone.0292455.s003.docx]
